# Temperature fluctuations in a warmer environment: impacts on microbial plankton

**DOI:** 10.12703/r/10-9

**Published:** 2021-01-29

**Authors:** Marco J Cabrerizo, Emilio Marañón

**Affiliations:** 1 Departamento de Ecología y Biología Animal, Universidade de Vigo, Facultad de Ciencias del Mar, Campus Lagoas Marcosende s/n, 36310 Vigo, Spain; 2 Centro de Investigación Mariña da Universidade de Vigo (CIM-UVigo), Illa de Toralla s/n, 36331, Vigo, Spain

**Keywords:** Aquatic ecosystems, global change, interactive effects, natural variability, thermal dependence

## Abstract

Warming can cause changes in the structure and functioning of microbial food webs. Experimental studies quantifying such impacts on microbial plankton have tended to consider constant temperature conditions. However, Jensen’s inequality (or the fallacy of the average) recognizes that organism performance under constant conditions is seldom equal to the mean performance under variable conditions, highlighting the need to consider *in situ* fluctuations over a range of time scales. Here we review some of the available evidence on how warming effects on the abundance, diversity, and metabolism of microbial plankton are altered when temperature fluctuations are considered. We found that fluctuating temperatures may accentuate warming-mediated reductions in phytoplankton evenness and gross photosynthesis while synergistically increasing phytoplankton growth. Also, fluctuating temperatures have been shown to reduce the positive warming effect on cyanobacterial biomass production and recruitment and to reverse a warming effect on cellular nutrient quotas. Other reports have shown that fluctuations in temperature did not alter plankton responses to constant warming. These investigations have mostly focused on a few phytoplankton species (i.e. diatoms and haptophytes) in temperate and marine ecosystems and considered short-term and transient responses. It remains unknown whether the same responses apply to other species and ecosystems and if evolutionary change in thermally varying environments could alter the magnitude and direction of the responses to warming observed over short-term scales. Thus, future research efforts should address the role of fluctuations in environmental drivers. We stress the need to study responses over different biological organization and trophic levels, nutritional modes, temporal scales, and ecosystem types.

## Introduction and context

Microbial plankton constitute the basis of the food web in most aquatic ecosystems and play a major role in element cycling, productivity, and the regulation of atmospheric CO_2_ levels^1^. Research efforts developed over the last few decades to understand how microbial plankton respond to warming have focused on large-scale averages across time^2^. However, environmental heterogeneity should be included as a target driver in biological manipulation experiments to obtain more realistic predictions of global warming impacts^3,4^.

## Effects of constant warming on aquatic ecosystems and organisms

**Figure 1.**
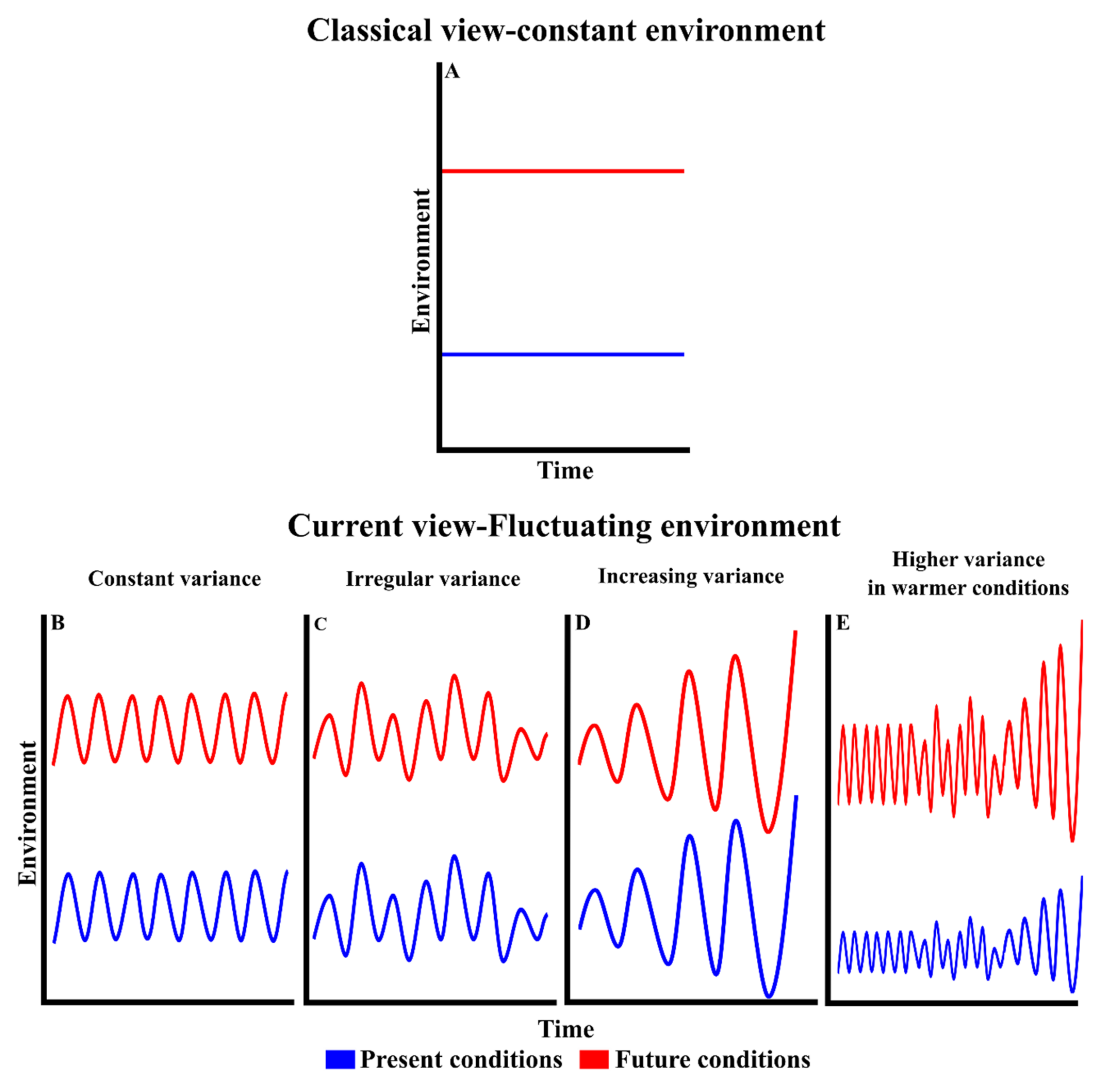
The classical and current view to evaluate the effects of global warming through experimental approaches.

Temperature governs all biochemical reactions^5^. Through its effect on metabolic rates, temperature has multiple repercussions on different biological organization levels, from populations to ecosystems^6^. For instance, warming stimulates preferentially heterotrophic versus autotrophic growth because of their higher thermal dependence^7^, and herbivorous protists’ growth compared with that of phototrophs^8^. It also promotes changes toward small-size protist communities when nutrients are limiting^9^ and can lead to losses of species richness and evenness in temperate phytoplankton communities^10^. Studies with experimental microbial food webs have shown that warming increases heterotrophic bacteria standing stocks and accelerates viral dynamics^11^, anticipates spring phytoplankton bloom timing, extending its duration^12^, and increases primary productivity^13^ but reduces their carbon sink capacity^14^. Finally, warming can alter the trophic interactions, including those of producer–consumer^15,16^ and host–parasite^17^, and reduce the efficiency of energy transfer to higher trophic levels^18^. Although most investigations have considered the effects of constant *in situ* or increased temperatures only (Figure 1A), already more than a century ago, Jensen^19^ stated through his famous inequality, also termed the fallacy of the average^20^, that the response of a system to constant average conditions is different from its mean response to variable conditions. Environmental variability can affect the response of communities and ecosystems to global warming through thermal fluctuations above and below mean temperatures, in which variance remains constant (Figure 1B) or is irregular (Figure 1C), and through amplified thermal fluctuations in which the variance increases over time (Figure 1D) or is higher in future respect to present conditions (Figure 1E), thus exposing organisms to more extreme conditions. Because of underlying non-linear relationships, thermal variability can improve or reduce performance compared to that predicted by thermal response curves built under constant conditions^20,21^. This prediction is supported by the observation that rate measurements at constant temperatures may overestimate or underestimate those rates occurring in naturally fluctuating environments^22,23^.

## Effects of fluctuating temperatures on aquatic ecosystems and organisms

Advances in remote sensing technologies and measurements from instruments anchored to floats, ocean gliders, and ships provide increasing evidence that surface ocean waters are a dynamic thermal environment, with temperatures fluctuating over varying time scales from changing weather, diel cycles, and ocean–atmosphere oscillations^24^. Moreover, these natural fluctuation patterns are being altered by climate change. For example, interannual variance has risen by more than 25% since 1980 in some areas (e.g. Europe)^25^, mainly due to an increasing occurrence of regional heatwaves since 1950^26^. Under this scenario, organisms are already experiencing abrupt shifts in their local temperature environment over short-term (from hours to weeks) and mid-term (seasons) scales rather than changes in climate per se, although long-term changes ultimately drive shorter ones^4^. Doblin and van Sebile^27^ demonstrated that this temperature variability can be up to 10°C greater than seasonal fluctuations estimated in a constant environment and that this variability depends strongly on location. Organisms naturally experiencing variations in temperature will tend to be generalists (i.e. highly plastic), having broad thermal breadths, whereas those from “stable” environments will likely be thermal specialists and will be restricted to specific regions/areas^28^. In addition, differences in generation times in populations can promote different adaptive dynamics to highly variable environments. This directional selection seems to be more effective for faster-growing than slower-growing populations because faster-growing microbes experience the “selective” environment for a larger number of generations^29^. These contrasting strategies may allow organisms adapted to fluctuating environments to grow faster, attain higher yield, or use resources more efficiently^30,31^. By contrast, it has also been proposed that increased temperature variation may pose a greater risk to species than the impacts derived from climate warming itself^32,33^. Bernhardt *et al.*^34^ found that fluctuating temperatures may reduce phytoplankton maximum growth rates by ~20%, their optimal temperature by ~3°C, and the maximum mean temperatures for positive growth by 2°C. Qu *et al*.^35^ reported reductions in specific nitrogen and carbon fixation rates in the nitrogen fixer *Trichodesmium* when compared to constant temperature conditions.

Zhang *et al.*^36^ have shown that increases in thermal variability have anticipated the cyanobacterial bloom initiation by ~80 days over the last three decades in Lake Taihu. Additionally, these authors have reported that cyanobacterial growth^36^ and photochemical performance^37^ are less sensitive to fluctuating temperatures than those of green algae and diatoms. Fluctuating temperatures can also promote both predator collapse^38^ and species competitive success, potentially facilitating biological invasions^39^, particularly when native species are not adapted to the fluctuating environment considered^40^. The underlying mechanism underpinning such observations is that directional selection on plasticity can also be weak, non-significant, or absent^41^, likely because production and maintenance costs can become too high to cover the increasingly wide environmental gradient that an organism experiences^21^. Therefore, ignoring the effects of environmental variability may limit our ability to predict how organisms are responding to ongoing warming, in particular those living at the edge of their thermal ranges.

## Interactions between warming and fluctuating temperature

Most laboratory investigations evaluating how fluctuating temperatures and warming interact have so far concentrated on a few well-studied species such as the coccolithophore *Emiliania huxleyi*^42^ and the diatom *Thalassiosira pseudonana*^43^. A deeper understanding about how populations respond to these drivers would entail knowing whether such responses can be extrapolated to other phytoplankton groups (e.g. cyanobacteria and dinoflagellates) and to other trophic levels (i.e. decomposers and grazers), even to similar species but with contrasting nutrition modes (i.e. mixotrophs versus strict phototrophs or heterotrophs). At the community level, most of the available evidence has focused on phytoplankton, while more comprehensive investigations at the ecosystem level (e.g. carbon sink capacity) are lacking.

The studies performed have considered either short-term scales (i.e. days), which represent acute/stress responses to the environmental drivers assayed^42,44^, or mid-term scales, that is, those that allow organisms’ acclimation^43,45,46^ (Table 1). Evolutionary responses over longer time scales to the interacting effect of warming and fluctuating temperature are still underrepresented^47^; however, it is known that thermal adaptation mediated by trait selection during evolutionary change can reverse short- and mid-term effects of constant warming on metabolic rates^48^.

**Table 1.**
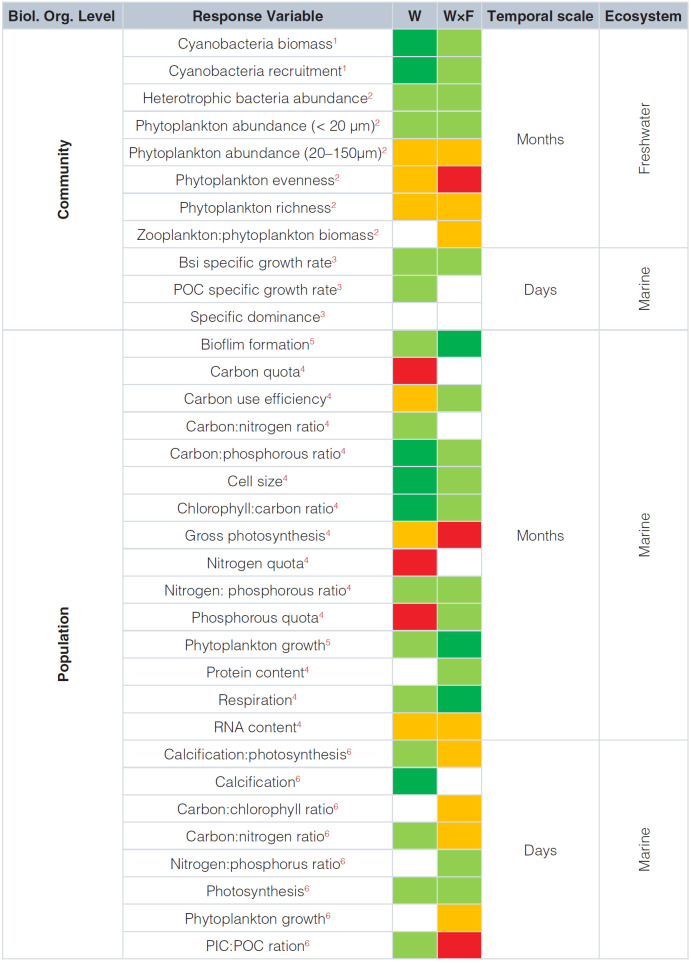
Qualitative effects of warming (W) and warming under fluctuating temperatures (W×F) on microbial plankton properties measured over different biological organization levels, temporal scales, and ecosystems. Rectangles represent absence of effect (white), negative effect (orange), strongly negative effect (red), positive effect (light green), and strongly positive effect (green). Superscript numbers in response variables represent studies where interactive effects of temperature fluctuations and warming were tested. Sources are ^1^Urrutia-Cordero *et al.*^50^, ^2^Rasconi *et al.*^45^, ^3^Kling *et al.*^44^, ^4^Schaum *et al.*^43^, ^5^Schaum *et al.*^46^ and ^6^Wang *et al.*^42^. Bsi represents biogenic silica, a proxy for diatom-specific rates, and PIC and POC are particulate inorganic and organic carbon, respectively.

There seems to be an imbalance between the amount of work conducted in different biomes, with marine ecosystems receiving more attention than freshwater environments. Although the ocean biome covers >75% of the Earth’s surface and its role in biogeochemical cycling is dominant, freshwater ecosystems, such as lakes and shallow ponds, have characteristics that also make them significant for global budgets. For example, these ecosystems exchange carbon at areal rates that are orders of magnitude greater than virtually any other global ecosystem (i.e. little things mean a lot)^49^.

Results available from experimental studies have been mostly performed in temperate areas (or species)^42,44,45,50^, whereas studies addressing the role of temperature fluctuations on microbial plankton in boreal/polar and tropical areas are scarce. Because thermal variability increases towards the poles^51^ and some high-latitude regions such as the Arctic are warming faster than the global average^52^, it becomes crucial to understand how the interplay between interacting environmental drivers modulates community responses in different biomes.

Temperature fluctuations have been shown to accentuate, attenuate, and even reverse the effect of warming on different properties and processes at the population and community level (Table 1). For instance, researchers have found a negative synergistic effect of the warming × fluctuating temperature interaction on gross photosynthesis^43^ and phytoplankton evenness^45^ but also a positive synergistic effect on microbial biofilm formation and phytoplankton growth^46^. Other reports indicate that temperature fluctuations can attenuate the positive effect of warming on cyanobacterial biomass production and recruitment^50^ or reverse the warming effect on carbon and nitrogen quotas^43^. Absence of effects has also been reported. For example, fluctuation in temperature did not affect species dominance in phytoplankton communities under warming conditions^44^ or alter the stimulatory effect of warming on photosynthetic activity in the coccolithophore *E. huxleyi*^42^.

The temperature dependence of plankton metabolic rates can be suppressed when nutrients are strongly limiting, hence some of the temperature effects discussed above might be circumscribed to ecosystems with high nutrient supply (e.g. coasts and upwelling systems)^53^. The interaction between nutrient availability and temperature variability is proving relevant to understand the dynamics and trophic functioning of microbial plankton communities. Model simulations and observations in tropical and temperate ecosystems show that phytoplankton blooms during heatwaves are weaker in nutrient-limited ecosystems and stronger when nutrients are high^54^. Nutrient limitation can weaken the producer–consumer interaction under warming conditions^15^ and increase the thermal range where a species is successful with respect to its competitors^55^.

## Future research directions

Despite the difficulties in testing and understanding how climate change affects microbial food webs because multiple environmental drivers are acting simultaneously^56^, future research efforts should take into account natural variations above and below mean trends in environmental drivers because these fluctuations could increase in frequency and intensity owing to the ongoing global warming. We stress the need to quantify these impacts over different biological organization levels (from molecules to ecosystems), different temporal scales (short versus long term), and types of ecosystems (marine and freshwater) to obtain a more comprehensive understanding of the magnitude and direction of global warming impacts on aquatic ecosystems.
